# CSF1R methylation is a key regulatory mechanism of tumor-associated macrophages in hepatocellular carcinoma

**DOI:** 10.3892/ol.2020.11726

**Published:** 2020-06-11

**Authors:** Bin Cui, Xiaoxiao Fan, Daizhan Zhou, Lifeng He, Yirun Li, Dandan Li, Hui Lin

**Affiliations:** 1 China-UK-NYNU-RRes Joint Laboratory of Insect Biology, Henan Key Laboratory of Insect Biology in Funiu Mountain, Nanyang Normal University, Nanyang, Henan 473061, P.R. China; 2 Department of General Surgery, Sir Run Run Shaw Hospital, School of Medicine, Zhejiang University, Hangzhou, Zhejiang 310016, P.R. China; 3 Biomedical Research Center, Sir Run Run Shaw Hospital, School of Medicine, Zhejiang University, Hangzhou, Zhejiang 310016, P.R. China

**Keywords:** colony stimulating factor 1 receptor, tumor-associated macrophages, microenvironment, hepatocellular carcinoma, methylation

## Abstract

Tumor-associated macrophages (TAMs) are important in tumor microenvironments and are closely associated with cancer occurrence, metastasis and progression. Colony stimulating factor 1 receptor (CSF1R) serves a crucial role in TAM formation. Whether CSF1R expression is regulated by DNA methylation in hepatocellular carcinoma (HCC) has not been fully elucidated. In the current study, HCC and adjacent non-cancerous tissue (ANT) samples were collected from 160 patients with HCC. CSF1R methylation levels were analyzed using a Mass ARRAY Analyzer to establish the potential impact of CSF1R methylation alternations on HCC clinicopathological characteristics. The mean methylation level of the CSF1R promoter (chr 5:149492491-149492958) was demonstrated to be significantly higher in ANTs compared with HCC tissues (65.3±7.5% vs. 57.3±14.4%, respectively; P<0.0001). CSF1R also exhibited decreased expression in HCC tissues compared with ANTs (P=0.0026). However, CSF1R expression was negatively correlated with CSF1R methylation levels in ANTs (r>0.4; P<0.0001). Further analysis indicated that patients with diabetes exhibited lower methylation levels in ANTs compared with HCC tissues (P=0.0062). Furthermore, CSF1R hypomethylation in ANTs was associated with a larger number of tumors (P=0.0332), larger tumor size (P=0.0494) and higher tumor grade (P=0.0244). Therefore, methylation alternation of the CSF1R promoter region analyzed in the present study was a key regulatory mechanism on CSF1R expression and ANT hypomethylation indicated poor clinicopathological characteristics of HCC. CSF1R may be a potential immunological therapeutic target for HCC.

## Introduction

Tumor microenvironments (TMEs) are a complex ecology of cells, comprising cancer-associated fibroblasts and various infiltrating immune cells that provide support to tumor cells during their transition to malignancy, such as tumor-associated macrophages (TAMs) (1). TAMs are a major constituent among the various innate and adaptive immune cells involved with the TME (2). TAMs are closely associated with tumor proliferation and metastasis and have been demonstrated to promote tumor angiogenesis, cancer cell infiltration into the circulation and suppression of antitumor immune mechanisms (3). Due to this involvement, TAMs are considered to be potential therapy targets in cancer treatment (4). However, the regulatory mechanisms underlying TAMs are yet to be fully elucidated and requires further research.

TAMs express cytokines and chemokines that suppress antitumor immunity (5). During the transition from benign growth to invasive tumor, colony stimulating factor-1 (CSF1) has been reported to be one of the key cytokines that regulates cancer-initiated inflammatory responses (6). CSF1 is a major lineage regulator of numerous macrophage populations, exerting its effect by controlling their production, differentiation and function (7). High CSF1 concentrations in tumors have been reported to be associated with poor prognosis (8). Furthermore, CSF1 and its receptor (CSF1R) have been reported to be central to the promotion of migration, survival and proliferation of monocytes (9).

CSF1R belongs to the platelet-derived growth factor receptor family and is a type III protein tyrosine kinase receptor (10). In addition to CSF1, CSF1R may also be recognized by other ligands, such as interleukin 34, leading to the full activation of the receptor (11). CSF1R-expressing macrophages have been reported to be associated with poor survival in various types of tumors, such as chronic lymphocytic leukemia and gastric cancer (12,13). Macrophages in tumor-infiltrating areas can selectively ignore the presence of tumor cells by highly expressing CSF1R (14). Additionally, a group of CSF1R-targeting small molecules and monoclonal antibodies have been revealed to be effective in mono- or combination-therapy (15).

In hepatocellular carcinoma (HCC), CSF1/CSF1R blockade has been reported to serve a critical role in the immunosuppressive nature of TMEs (16). CSF1R expression in macrophages serves a pivotal role in the interaction between macrophages and hepatoma cells (17). However, CSF1R expression and its potential regulatory mechanism in HCC requires further investigation.

The effect of methylation factors has been an area of interest in tumor research, and DNA methylation is reported to be a key regulatory mechanism in HCC (18). Previous epigenetics studies have revealed an association between CSF1R methylation and tumor proliferation or migration (19,20). In common malignant testicular germ cell tumors, CSF1R hypomethylation has been reported to be associated with poorer prognoses (21). Additionally, CSF1R expression was significantly elevated when demethylated, resulting in tumor metastasis promotion in melanoma (22). The results of the aforementioned studies have indicated that the CSF1R-mediated methylation regulatory mechanism served an important role in tumor development.

Therefore, in the present study, 160 adjacent non-cancerous tissue (ANT) and paired HCC tissue samples were collected, and methylation genotyping of the CSF1R promoter region was performed. The aim was to identify the expression and methylation alterations between HCC and normal tissues. As an important microenvironmental factor, the correlation between the methylation of the CSF1R promoter region and the clinicopathological features of the patients were analysed. Finally, the significance of methylation of the CSF1R promoter region in ANTs was explored for the early detection and treatment of HCC.

## Materials and methods

### 

#### Patients and tissue samples

The current study was approved by the Ethics Committee of the Department of Hepatobiliary Surgery at Sir Run Run Shaw Hospital and Zhejiang Hospital. All patients with HCC provided written informed consent. A total of 160 HCC samples and paired ANT samples (3 cm from the tumor) were collected from patients who underwent surgical liver tumor resection between July 2008 and February 2014. The samples were immediately frozen in liquid nitrogen and then stored at −80°C for DNA/RNA extraction. Pathological diagnosis was based on the morphological and immunohistochemical criteria provided by the World Health Organization (23). The tumor stages were classified according to the AJCC tumor-node-metastasis staging system (24).

Of the 160 patients with HCC, 111 were treated at Zhejiang Cancer Hospital and 49 at Sir Run Run Shaw Hospital. Their ages ranged between 31 and 76 years (mean age ± SD, 52.6±9.9 years), and the male-to-female ratio was 131:29. A total of 136 patients were positive for hepatitis B (HBV) and 53 patients were ≥TII according to the tumor-node-metastasis staging system. Detailed clinical information is summarized in Table I.

#### DNA extraction and bisulfite conversion

Total DNA was extracted from 25 mg tissue (both ANTs and tumor tissues) using a QIAamp^®^ DNA mini kit (Qiagen GmbH), according to the manufacturer's protocol. The DNA concentration was determined using a NanoDrop 2000 (Thermo Fisher Scientific, Inc.). Subsequently, a total of 500 ng DNA from each sample was modified by sodium bisulfite using the EpiTect Fast DNA Bisulfite kit (Qiagen GmbH) according to the manufacturer's protocol.

#### Gene bioinformatics and Sequenom analysis

CSF1R sequences were obtained from the human reference genome (GRch37/hg19; http://genome.ucsc.edu/) and were utilized to design the methylation genotyping primers using the online EpiDesigner software (www.epidesigner.com). For amplification of CSF1R from the DNA extracted from ANTs and tumor tissues, the following PCR primer pair was used: Forward, 5′AGGAAGAGAGTTTAGAGAGAGTAAGGGAGGGGTTA-3′ and reverse, 5′-CAGTAATACGACTCACTATAGGGAGAAGGCTTCATAATCAAACCCCAAATAAAAAA-3′. PCR was performed using the GeneAmp 9700 system (Applied Biosystems; Thermo Fisher Scientific, Inc.) in a 10-µl reaction containing 2 µl bisulfite-converted DNA (~10 ng/µl), 0.08 µl PCR enzyme (5 U/µl), 2 µl of each primer (1 µM), 1 µl PCR buffer (10×; Sequenom) and 0.08 µl deoxyribonucleotide triphosphates mix (25 mM each). The amplification process began with an initial 4-min denaturation at 94°C, followed by 45 cycles of 20 sec at 94°C, 30 sec at 56°C and 1 min at 72°C, and finally an extension step at 72°C for 3 min. Subsequently, the PCR products were treated with three standard procedures of the MassARRAY EpiTYPER (Sequenom), according to the manufacturer's protocol: Shrimp Alkaline Phosphatase cleanup, T cleavage and clean resin. Finally, the treated DNA was transferred to a MassARRAY Analyzer 4 (Sequenom) to analyze CSF1R promoter methylation, according to the manufacturer's protocol.

An in-house RNA sequencing (RNA-seq) dataset, which included data from tumor and ANTs of 11 patients with HCC, was established as previously described and was used to investigate the alteration of CSF1R expression between HCC tissues and ANTs (25). Additionally, the methylation and expression levels of CSF1R in 50 paired samples of patients with HCC were downloaded from The Cancer Genome Atlas (TCGA) database (https://portal.gdc.cancer.gov/) for validation. A total of 17 CpG probes in the CSF1R gene included in the Illumina HumanMethylation450 array from the TCGA database were analyzed, and 14 probes were successfully genotyped, but the methylation data of the remaining 3 probes were not available.

#### Immunohistochemical analysis

16 pairs (10% of patients with HCC) of ANTs and tumor tissues were randomly selected from the 160 patients with HCC to examine CSF1R protein expression via staining. Tissues used for immunohistochemistry were fixed in 10% neutral formalin at room temperature, embedded in paraffin and cut into 3-µm-thick sections. For staining, the sections were deparaffinized in xylene and rehydrated in a descending ethanol series (100, 95, 90, 80 and 70%, sequentially), and washed in water at room temperature. Antigen retrieval was performed using 0.01 M citrate buffer (pH 6.0) at high temperature in a pressure cooker for 5 min. Subsequently, the sections were treated with 3% hydrogen peroxide (diluted with methanol) for 10 min at 20°C and then incubated with 10% bovine serum albumin (cat. no. A8010; Beijing Solarbio Science & Technology Co., Ltd.) at room temperature for 30 min to block non-specific antibody binding. The slides were further incubated overnight with CSF1R antibody (1:100; cat no. ab52864; Abcam) at 4°C and then incubated with biotinylated secondary antibodies (ready to use; cat. no. GK600710/100; Gene Tech Biotechnology Co., Ltd.) for 30 min at room temperature. After light counterstaining with hematoxylin (cat. no. MB9897; Dailan Meilun Biology Technology Co., Ltd.) for 2 min at room temperature, the slides were dehydrated in an ascending ethanol series (70, 80, 90, 95 and 100%, sequentially), mounted with a coverslip and observed under a light microscope (magnification, ×100 and ×400; Nikon Eclipse 80i; Nikon Corporation).

Immunostaining was scored according to the German immunoreactive score (26). This 13-point method is used to determine the percentage of positive cells by assigning them 0–4 points: 0, no positive cells; 1, <10% positive cells; 2, 10–50% positive cells; 3, 50–80% positive cells; and 4, >80% positive cells. Staining intensity was graded as follows: 0, negative; 1, weak; 2, moderate; and 3, strong. The final score was calculated as the multiplication of these two indicators and ranged between 0 and 12. The bioinformatic analysis software TBtools (v0.67361) was used to generate the heat-maps (27).

#### Statistical analysis

SPSS software (version 25.0; IBM Corp.) was utilized in the present study for statistical analysis. The continuous type of clinical characteristics and methylation levels of each CpG sites were presented as the mean ± SD, and the categorical type of clinical characteristics were presented as number and percentage. Statistical differences in CSF1R methylation, RNA expression and HCC protein levels between tumor and paired ANT samples were analyzed using a paired t-test. Correlation between methylation and expression was analyzed by Pearson's correlation coefficient analysis. Step-wise linear regression was performed to investigate the association between clinicopathological characteristics and methylation levels in ANTs. Unpaired t-test and one-way ANOVA with the Scheffe post hoc test were used to evaluate methylation difference between binary variables (such as diabetes status, number of primary tumors and tumor stages) or multiple group variables (tumor size), respectively. Receiver operating characteristic (ROC) curves and the area under the curve (AUC) were utilized to evaluate if CSF1R methylation can be used as a predictor of biomarkers. P<0.05 was considered to indicate a statistically significant difference.

## Results

### 

#### Clinicopathological characteristics

Clinicopathological characteristics of 160 patients with HCC are detailed in Table I. Of those, 131 were male, 55 had a drinking habit (>50 g/day), 20 had diabetes and 37 had hyperlipidemia. Additionally, there were 104 patients with α fetoprotein (AFP) scores of >20 and 136 had HBV. A total of 142 patients exhibited cirrhosis.

#### CSF1R methylation level analysis

**Figure 1.**
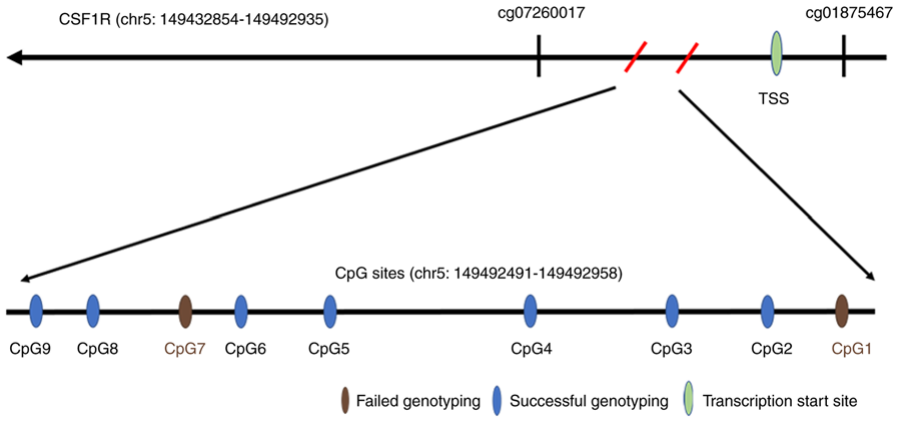
Image of the CSF1R gene fragment region with the locations of the selected CpG island regions and CpG sites. cg07260017 and cg01875467 are two probes from the Illumina HumanMethylation450 array used next to the target amplicon of CSF1R in the present study. CSF1R, colony stimulating factor-1 receptor; TSS, transcription start sites; chr, chromosome.

The CSF1R gene is located on chr5:149432854-149492935, and the target amplicon for methylation analysis was located on chr5:149492491-149492958. Therefore, the 468 bp amplicon ranged from −23 to 445 bp of the CSF1R gene, and only included the transcription start sites. A total of 9 CpG sites (sequentially named CpG1-9) were included in this amplicon region, but 2 sites (CpG1 and CpG7) failed to be detected due to limitations of the MassARRAY EpiTYPER technology (Fig. 1, Table SI). The methylation levels of 7 successfully genotyped CpG sites (CpG2, 3, 4, 5, 6, 8 and 9) demonstrated significant correlation with each other in both HCC and ANTs (Tables SII and SIII).

**Figure 2.**
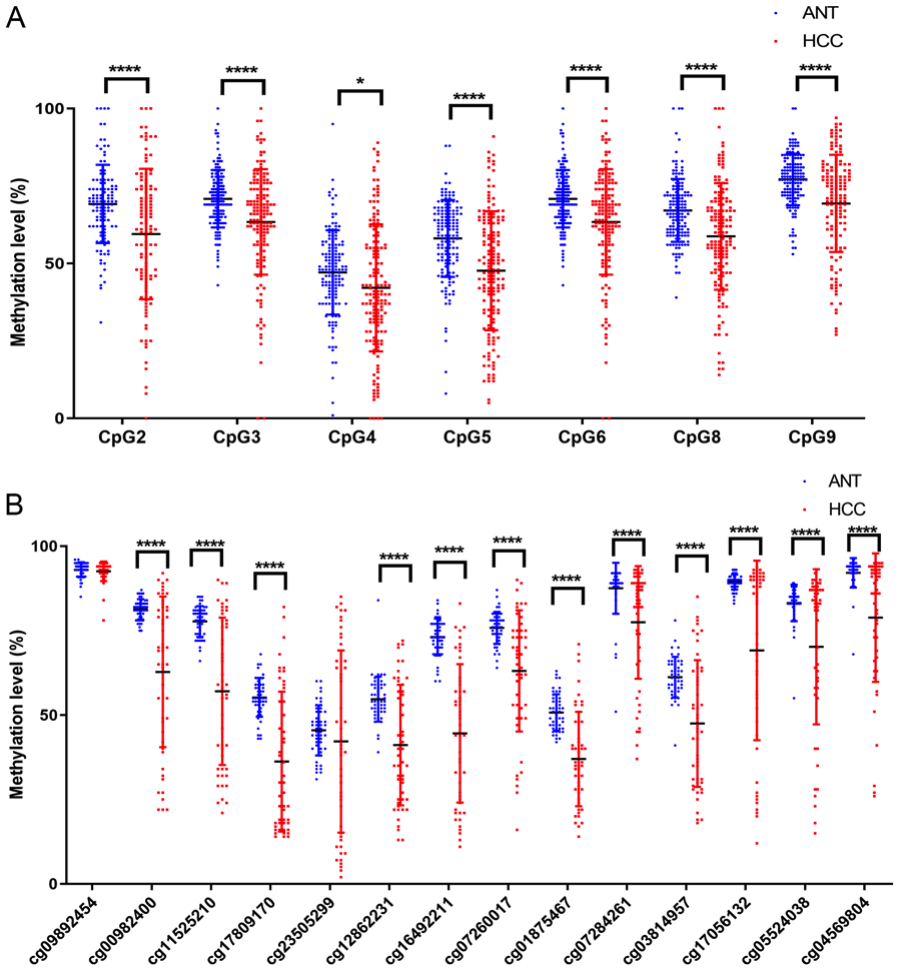
CSF1R methylation levels of ANT and HCC tissues analyzed by paired t-test. (A) Mean CSF1R methylation levels were significantly decreased in 160 HCC tissues compared with ANTs. (B) Mean CSF1R methylation levels were decreased in HCC tissues compared with ANT according to the data obtained from The Cancer Genome Atlas database. CpG1 and CpG7 detection failed for due to limitations of the MassARRAY EpiTYPER technology. *P<0.05; ****P<0.0001. CSF1R, colony stimulating factor-1 receptor; ANT, adjacent non-cancerous tissue; HCC, hepatocellular carcinoma.

Subsequently, paired t-tests were used to analyze the methylation levels of each CpG site in HCC and normal tissues. The results demonstrated that CSF1R methylation levels in all 7 successfully analyzed CpG sites were significantly decreased in HCC compared with their paired ANT samples (Table II; Fig. 2A) and that the mean methylation difference between HCC and ANTs ranged between 4.9 and 11.0% in the 7 CpG sites (Table II). The mean methylation level in the CSF1R promoter was 57.3±14.4% in HCC tissues and 65.3±7.5% in ANTs, respectively (P<0.0001; Table II). Additionally, the present results were supported by data from TCGA database. The methylation data of CSF1R in 50 HCC tissues and their paired ANTs from TCGA (https://portal.gdc.cancer.gov/) database were assessed, and 12 CpG probes of the Illumina HumanMethylation450 array in the CSF1R gene were hypomethylated in HCC tissues (Fig. 2B).

**Figure 3.**
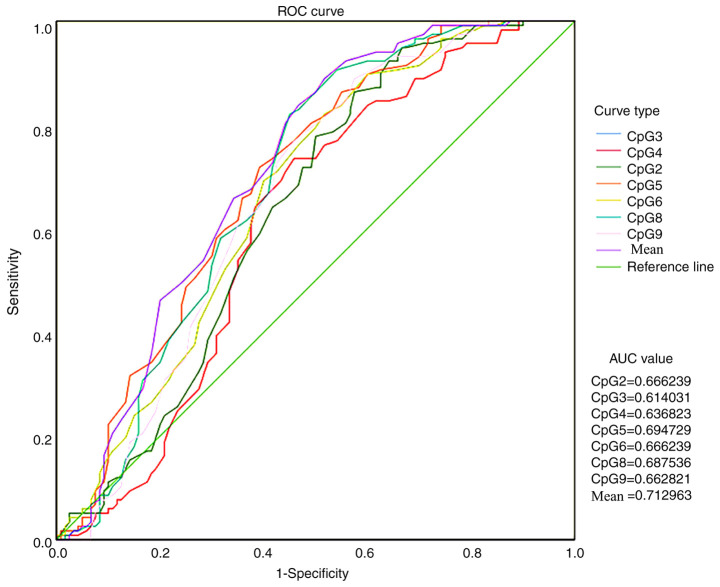
ROC curve analysis of CSF1R methylation levels. ROC, receiver operating characteristic; CSF1R, colony stimulating factor-1 receptor; AUC, area under the curve.

ROC curve analysis was conducted to compare CSF1R methylation levels in ANTs and HCC tissues and to verify whether the methylation level of the CSF1R promoter region could be used as a biomarker for HCC diagnosis and treatment (Fig. 3). All AUC values were >0.5 and the mean value of all methylation sites was 0.713.

**Figure 4.**
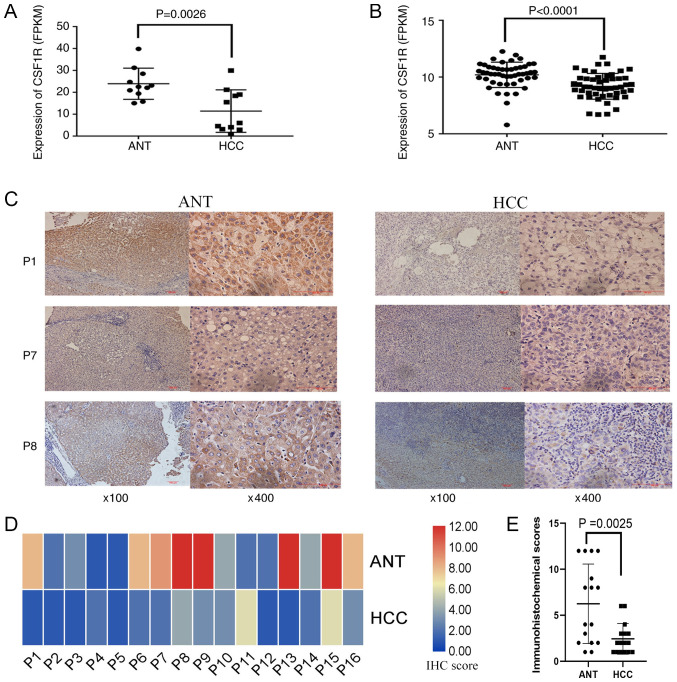
CSF1R expression in ANTs and HCC tissues. (A) CSF1R expression in 11 pairs of ANTs and HCC tissues from patients with HCC (P=0.0026; paired t-test). (B) CSF1R expression in 50 pairs of ANTs and tumor tissues from patients with HCC. Information was obtained from TCGA (P<0.0001; paired t-test). (C) Tissue sections of 3 patients with HCC (P1, P7 and P8). IHC images were captured under a magnification, ×100 and ×400 using a light microscope. (D) Heatmap of the IHC results from 16 patients with HCC scored according to the 13-point method of the German IHC scoring system. Red indicated high expression and blue indicated low expression. Color depth indicated the level of expression. (E) Difference of CSF1R IHC score in ANTs and HCC tissues of 16 patients with HCC (P=0.0025; paired t-test). CSF1R, colony stimulating factor-1 receptor; ANT, adjacent non-cancerous tissue; HCC, hepatocellular carcinoma; FPKM, reads per kilobase of exon model per million mapped reads; TCGA, The Cancer Genome Atlas; P1/7/8, patient 1/7/8; IHC, immunohistochemical.

To analyze the alteration of CSF1R expression in HCC, an in-house RNA-seq dataset containing 11 HCC tissues and their paired ANTs was used. In this dataset, a significantly higher CSF1R expression was detected in ANTs compared with HCC tissues [normal tissues vs. cancer tissues; reads per kilobase of exon model per million mapped reads (FPKM), 23.94 vs. 11.43; P=0.0026; Fig. 4A]. In addition, CSF1R expression was then examined from TCGA dataset, from which 50 paired HCC RNA-seq data were downloaded and analyzed using paired t-tests. The results from TCGA database revealed that CSF1R expression was significantly decreased in HCC tissues compared with that in ANTs (ANTs vs. HCC tissues; FPKM, 10.21 vs. 9.19; P<0.0001; Fig. 4B).

Furthermore, immunohistochemistry was performed to detect CSF1R protein expression in 16 paired HCC and ANTs, and to verify expression differences at the protein level. The results indicated that CSF1R protein levels in ANTs were markedly higher compared with HCC tissues (Fig. 4C), which was consistent with the data that was sequenced and obtained from TCGA database. Using the 13-point method, it was also suggested that CSF1R protein expression in ANTs was significantly higher compared with that in tumor tissues (P=0.0025; Fig. 4D and E).

**Figure 5.**
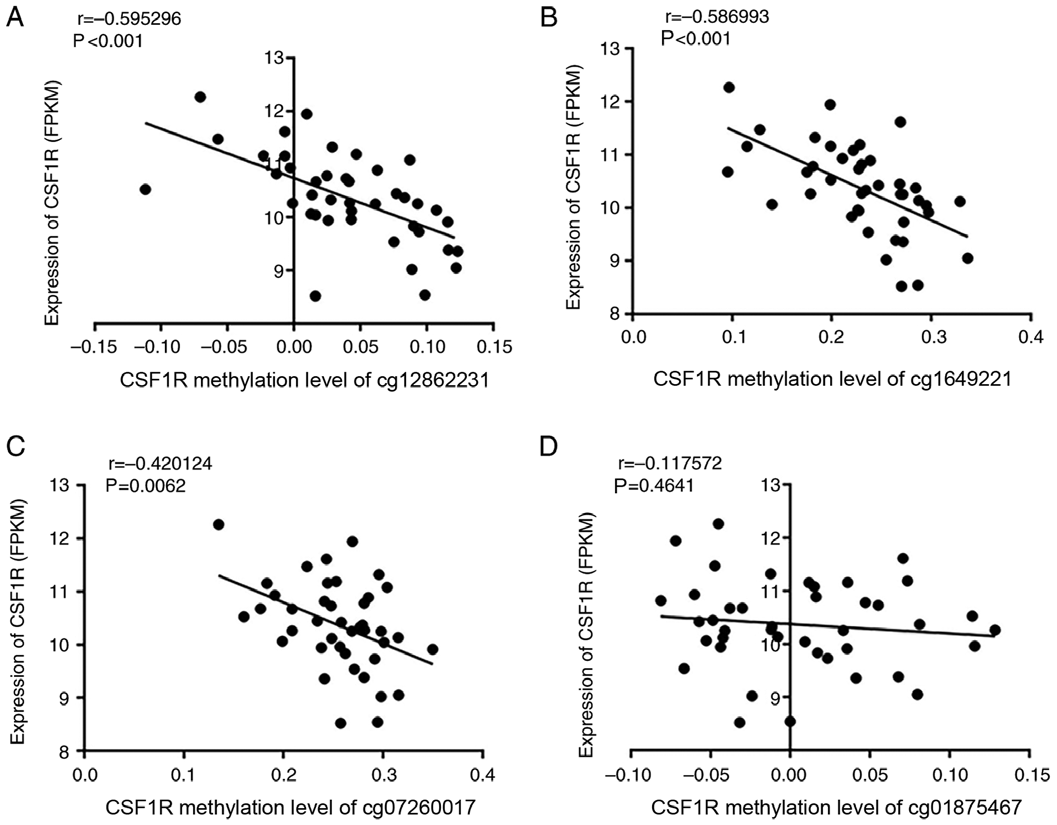
Correlation between the methylation of 4 sites near the transcription start sites and CSF1R RNA expression in adjacent non-cancerous tissues of 50 patients with HCC from The Cancer Genome Atlas database. The correlation was analyzed via Pearson's correlation coefficient analysis. CSF1R methylation levels of (A) cg12862231 (P<0.001; r=−0.595), (B) cg1649221 1 (P<0.001; r=−0.587), (C) cg07260017 (P=0.006; r=−0.420) and (D) cg01875467 (P=0.464; r=−0.118) results are presented. CSF1R, colony stimulating factor-1 receptor; FPKM, reads per kilobase of exon model per million mapped reads.

CSF1R expression and methylation data were downloaded from TCGA datasets to identify the correlation between the expression and methylation status. A significantly negative correlation was identified between methylation and expression of CSF1R in ANTs (Fig. 5A and B), particularly in sites cg12862231 and cg16492211 (P<0.001). Sites cg07260017 and cg01875467, which were adjacent to the selected CpG islands according to TCGA data, were also analyzed and revealed to follow the same trend (Fig. 5C and D). However, when combining the ANT and paired HCC data together, there was no correlation between CSF1R methylation and expression (Fig. SI).

#### Correlation between CSF1R promoter methylation status and patient clinicopathological characteristics

**Figure 6.**
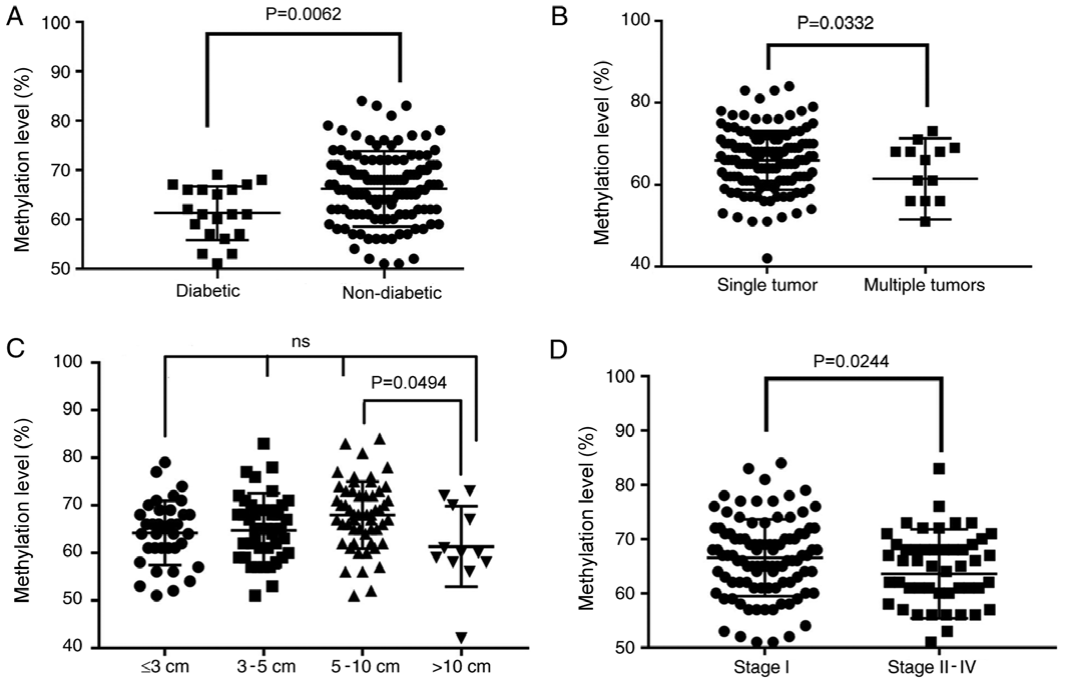
Association between four clinicopathological characteristics and CSF1R methylation levels in adjacent non-cancerous tissues of 160 patients with HCC. (A) Diabetic vs. non-diabetic (P=0.0062; unpaired t-test). (B) Single tumor vs. multiple tumors (P=0.0332; unpaired t-test). (C) Size of tumor (P=0.0494; one-way ANOVA). (D) Stage I vs. stage II–IV (P=0.0237; unpaired t-test). CSF1R, colony stimulating factor-1 receptor; ANT, adjacent non-cancerous tissue; ns, not significant.

To determine the potential effects of CSF1R methylation and expression in HCC, the correlation between the methylation status of CSF1R and comprehensive clinicopathological features was analyzed. According to the high correlation among the methylation levels of the CpG sites (Tables SII and SIII), the mean methylation levels of the 7 CpG sites were used to evaluate their association with clinicopathological characteristics. The results revealed that diabetes status was associated with methylation levels of CSF1R. The mean value of the methylation site in the ANTs of patients with diabetes was significantly different from that in tissues from patients without diabetes. Patients with HCC that concurrently presented with diabetes exhibited low ANT methylation (66.2% in 140 non-diabetic vs. 61.3% in 20 diabetic patients; P=0.0062; Fig. 6A and Table I).

Furthermore, the results demonstrated that patients with HCC who presented with multiple tumors exhibited an average methylation level that was significantly decreased compared with patients with single tumors (65.9 vs. 61.4%; P=0.0332; Fig. 6B and Table I). Additionally, there was a significant difference between tumor diameters of >10 cm and 5–10 cm (P=0.0494; Fig. 6C and Table I) as demonstrated by ANOVA analysis followed by the Scheffe post hoc test. However, a linear decrease with an increase in tumor diameter was not reported. This could be due to the current sample size being too small. Concurrently, the results revealed that the average ANT methylation levels of patients with high-stage HCC also exhibited a significant decrease (66.5 vs. 63.6%; P=0.0237; Fig. 6D and Table I).

## Discussion

The present study investigated whether CSF1R methylation levels in ANTs from patients with HCC had a regulatory effect on HCC progression and whether CSF1R methylation levels in ANTs could be used as clinical biomarkers for HCC. The target region of the CSF1R promoter includes 9 CpG sites, but 2 sites failed to be genotyped in the present study. The methylation levels of the remaining 7 CpG sites exhibited significant correlation among each other, and the mean methylation levels of these 7 CpG sites were used to analyze the association between CSF1R methylation and clinicopathological characteristics. Therefore, although 2 CpG sites in the CSF1R promoter were excluded from the present analysis, it is hypothesized that they would not have had a significant impact on the results of the present study.

By genotyping CSF1R methylation in ANTs and tumor tissues of patients with HCC, the level of methylation in ANTs was determined to be significantly higher compared with tumor tissues. CSF1R expression in ANTs was also revealed to be significantly higher than that of tumor tissues. However, methylation and expression of CSF1R in ANTs demonstrated a significant negative correlation as evidenced by HCC data from TCGA database. These results appear to be contradictory. In fact, the methylation levels of CSF1R in tumor tissues had a more discrete distribution than those in ANTs (Fig. 2). When combining the ANT and HCC data together, there was no correlation between CSF1R methylation and expression (Fig. SI). A potential reason may be the different percentages of immune cells between ANTs and HCC tissues. CSF1R promoter region methylation in ANTs may have a special regulatory pattern for CSF1R expression, and therefore further studies are required to confirm these results.

ROC curve analysis of ANT and tumor methylation data was conducted and revealed that CSF1R methylation had a potential role in differentiating between cancer and normal tissues. This indicated that the CSF1R methylation site in ANTs may be a possible biomarker for HCC diagnosis (28). In addition, several studies have indicated that TMEs are crucial in tumor progression and cancer treatment (29–31). CSF1R has been reported to serve an important regulatory role in TMEs (32–34). As the receptor for CSF1, CSF1R is activated after CSF1 binding and can regulate macrophage differentiation (35). It has been reported that high CSF1R expression in TMEs may cause the progression of TAMs into the M2 type, which results in the loss of macrophage immunity (36,37). Additionally, M2 type TAMs can promote malignant tumor progression (2). A previous study has reported that CSF1R inhibitors could be developed as novel potential anticancer compounds (9). Therefore, CSF1R seems to have a comprehensive clinical application value in HCC and should be further investigated in future studies.

In the present study, patients with HCC who also had diabetes exhibited significant ANT hypomethylation levels. Furthermore, immunohistochemical staining of tissue sections from patients with HCC demonstrated that CSF1R expression in ANTs was significantly higher compared with HCC tissues. The correlation between CSF1R methylation and expression in the TCGA database identified a significant negative correlation. These results indicated that there was an association between the methylation level of CSF1R in the ANTs of patients with HCC and diabetes, thereby regulating CSF1R expression. A previous study has demonstrated that diabetes is more likely to trigger tumor macrophages to promote colorectal cancer formation (38). Additionally, diabetes has been reported to be an important factor in the induction of liver cancer (39). Furthermore, numerous studies have revealed that diabetes is associated with the development of multiple types of cancer, such as liver and endometrial cancer (40,41). Therefore, the detection of CSF1R methylation levels may be a possible predictor of HCC in patients with diabetes. However, the specific regulatory relationship requires further experimental research.

Correlation analysis between ANT methylation data and clinicopathological characteristics revealed that the level of methylation in ANT sites was significantly reduced in patients with HCC exhibiting multiple tumors. When the tumor diameter was >10 cm, a significant decrease was observed in the average methylation level of the ANTs. No linear decline was observed in methylation level according to diameter growth. This may have been due to the insufficient experimental sample size. These results further demonstrated that lower methylation resulted in high CSF1R expression, which may be causing a decrease in the immunosuppressive function of TAMs and may be leading to tumor development and metastasis. Additionally, the level of CSF1R methylation in ANTs was significantly reduced in patients with advanced stage HCC. Lower methylation was accompanied by higher expression, indicating that there is a regulatory relationship between CSF1R hypomethylation in ANTs and tumor progression. Methylation levels of CSF1R in ANTs may therefore be utilized to predict tumor progression in patients with HCC.

In conclusion, the current study demonstrated that the methylation level of CSF1R in the ANTs from patients with HCC regulated TMEs, which serve a role in the regulation of metastasis. Methylation was a key regulatory mechanism of CSF1R expression, and CSF1R hypomethylation in ANTs was associated with poor clinicopathological characteristics of patients with HCC. Furthermore, CSF1R may be a potential immunological therapeutic target for HCC.

## Supplementary Material

Supporting Data

## Figures and Tables

**Table I.** Clinicopathological characteristics of 160 patients with hepatocellular carcinoma and their mean CSF1R methylation levels in adjacent non-cancerous tissues.

Parameter	No. of patients	Mean methylation level of CSF1R Mean levels (%) in ANTs	P-value^b^
Sex			
Female	29 (18.1%)	68.0±7.0	0.0756
Male	131 (81.9%)	65.0±7.6	
Age (years)			
≥55	79 (49.4%)	64.6±7.0	0.1652
<55	81 (50.6%)	66.4±7.9	
Alcohol habit^a^			
Yes	55 (34.4%)	65.0±8.2	0.4850
No	105 (65.6%)	65.9±7.2	
Hypertension			
Yes	37 (23.1%)	63.8±6.7	0.1115
No	123 (76.9%)	66.1±7.7	
Diabetes			
Yes	20 (12.5%)	61.3±5.4	0.0062
No	140 (87.5%)	66.2±7.6	
Hyperlipemia			
Yes	37 (23.1%)	63.8±6.6	0.1234
No	121 (75.6%)	66.0±7.8	
AFP (µg/l)			
<20	53 (33.1%)	64.7±6.4	0.2658
≥20	104 (65.0%)	66.1±8.0	
CEA (µg/l)			
≤5	132 (82.5%)	66.1±7.6	0.0639
>5	24 (15%)	63.1±6.5	
HBV			
Yes	136 (85%)	65.9±7.7	0.3296
No	22 (13.8%)	64.2±6.1	
No. of tumors			
<2	145 (90.6%)	65.9±7.2	0.0332
≥2	15 (9.4%)	61.4±9.9	
Tumor diameter (cm)			
≤3	38 (23.8%)	64.2±6.8	0.0101
3-5	48 (30%)	64.7±7.8	
5-10	60 (37.5%)	68.0±7.0	
>10	14 (8.8%)	61.3±8.4	
Capsule invasion			
Yes	108 (67.5%)	65.8±8.3	0.4167
No	52 (32.5%)	64.9±5.8	
Tumor necrosis			
Yes	29 (18.1%)	67.5±7.0	0.1227
No	131 (81.9%)	65.1±7.6	
Liver cirrhosis			
Yes	142 (88.8%)	65.3±7.8	0.1480
No	13 (8.1%)	66.4±5.3	
Microvascular invasion			
Yes	106 (66.3%)	66.2±7.8	0.1540
No	54 (33.8%)	64.3±6.9	
TNM stage			
I	106 (66.3%)	66.6±7.0	0.0244
≥II	53 (33.1%)	63.6±8.2	

a Patients with an alcohol habit drank >50 g/day of alcohol.

b P-values indicate the difference in methylation levels under different pathological features in ANTs. CSF1R, colony stimulating factor-1 receptor; HBV, hepatitis B virus; TNM, tumor node metastasis; AFP, αfetoprotein; CEA, carcinoembryonic antigen; ANTs, adjacent non-cancerous tissues.

**Table II.** CSF1R promoter methylation in patients with HCC.

		HCC		
		
CpG	Group	Mean (%)	ΔMean (%)^b^	P-value
CpG 2	ANT	68.2±12.1	10.3	<0.0001
	HCC	57.9±20.2		
CpG 3	ANT	70.8±9.4	7.4	<0.0001
	HCC	63.4±15.9		
CpG 4	ANT	46.7±13.2	4.9	0.0070
	HCC	41.8±19.5		
CpG 5	ANT	57.9±12.6	11.0	<0.0001
	HCC	46.9±18.3		
CpG 6	ANT	70.8±9.4	7.4	<0.0001
	HCC	63.4±15.9		
CpG 8	ANT	66.8±10.1	8.3	<0.0001
	HCC	58.5±16.5		
CpG 9	ANT	77.0±8.4	7.9	<0.0001
	HCC	69.1±15.5		
Mean^a^	ANT	65.3±7.5	8.1	<0.0001
	HCC	57.3±14.4		

a Mean methylation levels of the 7 CpG sites in the target amplicon of the CSF1R promoter.

b ΔMean represents the mean difference in methylation levels between ANTs and HCC tissues. ANT, adjacent non-cancerous tissue; CSF1R, colony stimulating factor-1 receptor; HCC, hepatocellular carcinoma; HBV, hepatitis B virus.

## Data Availability

The datasets used and/or analyzed during the present study are available from the corresponding author on reasonable request. Detailed pathological features and methylation data of patients are not available to the public.
