# Supplementary material for: CSF1R methylation is a key regulatory mechanism of tumor-associated macrophages in hepatocellular carcinoma
Source: Oncol Lett. 2020 Jun 11;20(2):1835–45. doi: 10.3892/ol.2020.11726 (PMC7377184; doi:10.3892/ol.2020.11726)
Supplement: Supporting Data [file Supplementary_Data.pdf]

Figure S1. Correlation between CSF1R methylation and expression in combined hepatocellular carcinoma and adjacent non-cancerous tissue samples. (A) cg12862231, (B) cg1649221, (C) cg07260017 and (D) cg01875467. CSF1R, colony stimulating factor 1 receptor.

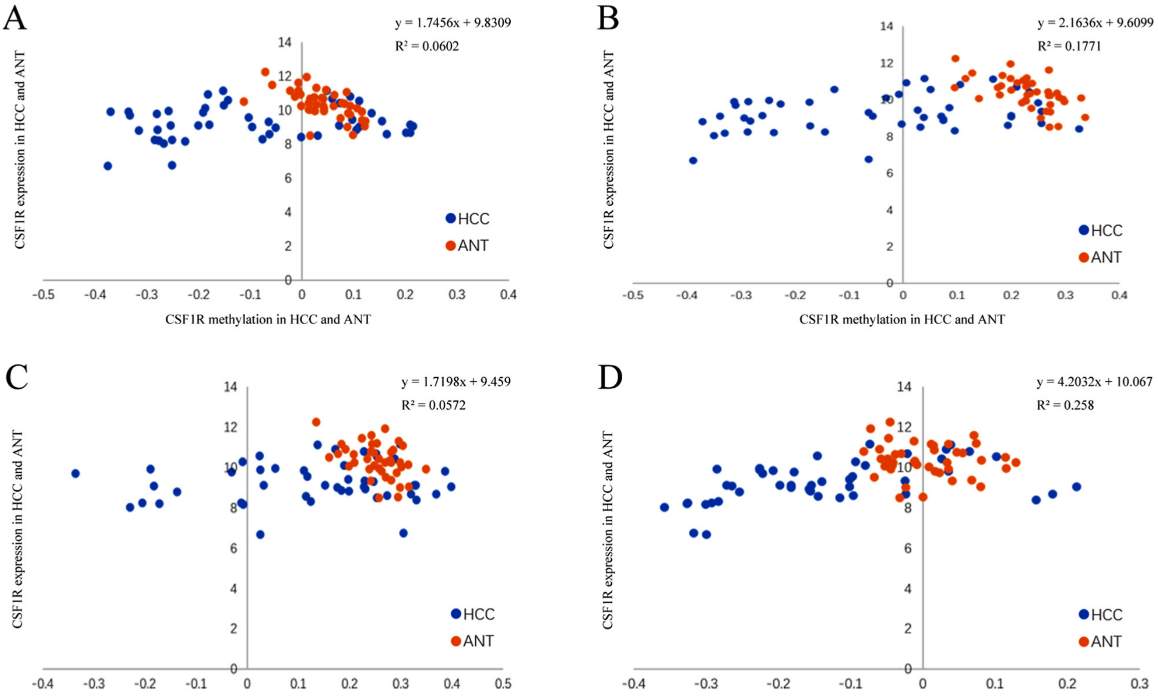

Table SI. Specific locations of each locus shown in Fig. 1.

Locus	Location
Transcription start site	chr5: 149492935
CpG1	chr5: 149492911
CpG2	chr5: 149492902
CpG3	chr5: 149492846
CpG4	chr5: 149492835
CpG5	chr5: 149492801
CpG6	chr5: 149492742
CpG7	chr5: 149492714
CpG8	chr5: 149492690
CpG9	chr5: 149492669
cg01875467	chr5: 149493008
cg07260017	chr5: 149492480

cg01875467 and cg07260017 represent the probe IDs in the Illumina Infinium HumanMethylation 450 array. chr, chromosome.

Table SII. Methylation correlation in 7 CpG sites of colony stimulating factor 1 receptor in tumor tissues.

		R						
		TCpG2	TCpG3	TCpG4	TCpG5	TCpG6	TCpG8	TCpG9
P-value	TCpG2	N/A	0.5621[a]	0.5203[a]	0.5916[a]	0.5621[a]	0.4535[a]	0.5100[a]
	TCpG3	<0.0001	N/A	0.6704[a]	0.7501[a]	1.0000[a]	0.6270[a]	0.5589[a]
	TCpG4	<0.0001	<0.0001	N/A	0.7637[a]	0.6704[a]	0.5424[a]	0.6483[a]
	TCpG5	<0.0001	<0.0001	<0.0001	N/A	0.7501[a]	0.4273[a]	0.5544[a]
	TCpG6	<0.0001	<0.0001	<0.0001	<0.0001	N/A	0.6270[a]	0.5589[a]
	TCpG8	<0.0001	<0.0001	<0.0001	<0.0001	<0.0001	N/A	0.8148[a]
	TCpG9	<0.0001	<0.0001	<0.0001	<0.0001	<0.0001	<0.0001	N/A

[a]P<0.01. TCpG, tumor CpG sites; N/A, not applicable.

Table SIII. Methylation correlation of 7 CpG sites of colony stimulating factor 1 receptor and adjacent non-cancerous tissues.

		R						
		NCpG2	NCpG3	NCpG4	NCpG5	NCpG6	NCpG8	NCpG9
P-value	NCpG2	N/A	0.1791	0.4138[b]	0.2079[a]	0.1791	0.3739[b]	0.3680[b]
	NCpG3	0.0533	N/A	0.4032[b]	0.3629[b]	1.0000[b]	0.2924[b]	0.3476[b]
	NCpG4	<0.0001	<0.0001	N/A	0.6300[b]	0.4032[b]	0.0917	0.3076[b]
	NCpG5	0.0245	<0.0001	<0.0001	N/A	0.3629[b]	0.0414	0.4232[b]
	NCpG6	0.0533	<0.0001	<0.0001	<0.0001	N/A	0.2924[b]	0.3476[b]
	NCpG8	<0.0001	0.0003	0.2662	0.6162	0.0003	N/A	0.5829[b]
	NCpG9	<0.0001	<0.0001	0.0001	<0.0001	<0.0001	<0.0001	N/A

[a]P<0.05; [b]P<0.01. NCpG, non-cancerous CpG sites; N/A, not applicable.
